# Monitoring Health Problems in Military Personnel Using the Oslo Sports Trauma Research Center Questionnaire on Health Problems

**DOI:** 10.1093/milmed/usag174

**Published:** 2026-04-30

**Authors:** Pablo M Stegerhoek, Jesse van der Zande, T Herman IJzerman, P Paul F M Kuijer, Evert A L M Verhagen

**Affiliations:** Department of Public and Occupational Health, Amsterdam Movement Sciences, Amsterdam UMC, Amsterdam Collaboration on Health and Safety in Sports, VU University Medical Center, Amsterdam 1081 BT, The Netherlands; Department of Public and Occupational Health, Amsterdam Public Health Research Institute, Amsterdam UMC, University of Amsterdam, Amsterdam 1105 AZ, The Netherlands; Health Care Section, Plein-Kalvermarkt-Complex, Royal Netherlands Marechaussee, Den Haag, 2511 CB, The Netherlands; Health Care Section, Plein-Kalvermarkt-Complex, Royal Netherlands Marechaussee, Den Haag, 2511 CB, The Netherlands; Health Care Section, Plein-Kalvermarkt-Complex, Royal Netherlands Marechaussee, Den Haag, 2511 CB, The Netherlands; Department of Public and Occupational Health, Amsterdam Movement Sciences, Amsterdam UMC, Amsterdam Collaboration on Health and Safety in Sports, VU University Medical Center, Amsterdam 1081 BT, The Netherlands; Department of Public and Occupational Health, Amsterdam Public Health Research Institute, Amsterdam UMC, University of Amsterdam, Amsterdam 1105 AZ, The Netherlands; Department of Public and Occupational Health, Amsterdam Movement Sciences, Amsterdam UMC, Amsterdam Collaboration on Health and Safety in Sports, VU University Medical Center, Amsterdam 1081 BT, The Netherlands

## Abstract

**Introduction:**

Military personnel are exposed to diverse, intense, and often unchangeable occupational demands, resulting in a high prevalence and burden of health problems. Traditional health registration methods, like medical records, only capture health problems when they result in limited duty status, often not allowing for timely intervention. Therefore, this study investigated the relationship between occupational tasks and the development of health problems at the Royal Netherlands Marechaussee (RNLM) using the Oslo Sports Trauma Research Centre Questionnaire on health problems (OSTRC-H2).

**Materials and Methods:**

This was a 12-week prospective observational cohort study at the operational branch of the RNLM. All about 6,500 employees of the RNLM were able to participate. Participants used the Perfyso app to register their health status and occupational tasks weekly. We used logistic mixed model analyses to estimate the effect of the occupational task on the occurrence of a health problem.

**Results:**

Three hundred forty-six participants registered to participate and 293 filled in the first week questionnaire with a mean weekly response rate of 51% (95% CI: 40%-61%). Participants reported a total of 224 health problems with a mean weekly prevalence of 23% (for all health problems) and 5% for substantial health problems. Most of the reported health problems were illnesses (43%), followed by physical health problems (35%), and mental health problems (22%). We found that dynamic and operationally demanding tasks, such as Border Policing—Mobile Security Surveillance and Security and Surveillance—Subjects, were associated with a higher risk of health problems compared to less demanding tasks like managing or deskwork.

**Conclusions:**

This study found that dynamic and operationally demanding tasks were associated with a higher risk of health problems compared to less demanding tasks. Our findings indicate that this tool may be further explored to monitoring emerging health problems in military personnel.

## INTRODUCTION

Military personnel must be physically and mentally capable of handling high and diverse occupational demands, such as carrying heavy protective equipment, staying alert during long shifts, or responding appropriately to dangerous situations. As a result, the prevalence and burden of work-related physical and mental health problems are high.^1^ For example, Hauret et al.^1^ reported an annual total of 1.6 million injuries in the U.S. Military, of which 743,547 involved nondeployed musculoskeletal injuries. Additionally, 5% to 16% of active duty military personnel reported a mental health problem predeployment and 11% to 29% postdeployment.^2^ The combination of high occupational demands and high incidence rates of adverse health problems poses a significant financial burden and a threat to military readiness.^3^

Underreporting of health problems is a widely recognized problem in both the civilian^4^ and military populations.^5^ In the military context specifically, only 51% of personnel with musculoskeletal injuries and 23% to 40% of personnel with mental health problems intended to seek medical care.^5^^,^^6^ Additionally, traditional health problem registration methods are based upon personnel seeking medical help and, therefore, cannot capture health problems that are perceived as not severe enough to report. Traditional registration methods only capture health problems when they result in limited duty status.^7^ Delayed identification can result in the deterioration of health problems that could have been mitigated had medical experts been aware of the problem.^5^

In the sports context, the Oslo Sports Trauma Research Centre Questionnaire for Health Problems (OSTRC-H2) is used to capture the severity and impact of a health problem on functioning, rather than only the resulting time loss.^8^ Mashimo et al.^9^ demonstrated that the OSTRC-H2 questionnaire increased the number of registered health problems threefold compared to traditional injury registration methods. Although typically used for sports-related injuries, van Winden et al.^10^ have used the OSTRC-H2 to register all health problems, including mental health problems, among contemporary dance students. Furthermore, Bjørneboe et al.^11^ found the OSTRC-H2 to be suitable for detecting health problems in the military context.

The Royal Netherlands Marechaussee (RNLM) is 1 of the 4 branches of the Dutch armed forces, responsible for safeguarding the security of the Kingdom of the Netherlands both domestically and internationally. In practice, this mandate encompasses a wide range of tasks, including the protection of high-risk objects and persons, border policing, and military police duties.^12^ These tasks vary substantially in their physical demands, ranging from predominantly static and sedentary activities, such as border control, to highly physically demanding operations involving prolonged load carriage of body armor and weaponry during security and military police tasks.

Given the high burden of health problems, the degree of underreporting, and the importance of identifying health problems before they result in limited duty status in the military context, it is paramount that surveillance tools that capture health problems early are investigated and if effective are implemented. Therefore, the current study examined the prevalence of physical and mental health problems in a Dutch military population using the OSTRC-H2 questionnaire to see if military personnel are willing to timely report these health problems. Additionally, given the apparent relationship between occupational demands and health problems in the military, this study investigated the relationship between these health problems and occupational tasks in RNLM personnel over a 12-week period.

## METHODS

### Design

This 12-week prospective observational cohort study included operational personnel from the RNLM. We used a mobile application (Perfyso) to distribute a weekly questionnaire regarding health problems and occupational tasks to participants each Friday. The Amsterdam UMC medical ethics committee approved this study (ref: 2023.0026), and all participants provided digital informed consent. We adhered to the Strengthening the Reporting of Observational Studies in Epidemiology (STROBE) guidelines in the reporting of our results.^13^

### Setting and Population

The RNLM, a branch of the Dutch military, has 3 branches: the staff, the Training and Education Centre (Dutch: OTC), and the National Tactical Command (Dutch: LTC).^12^ The LTC consists of 25 brigades located across the Kingdom of the Netherlands. Each brigade has a unique set of responsibilities and tasks. For example, one brigade at Schiphol Airport is responsible for border security, while another brigade in The Hague is responsible for protecting the National Parliament.

### Recruitment

All ∼6,500 LTC employees received a recruitment email from a dedicated study address, including basic procedural and study information, a link to a recruiting video, and an intranet page with further details. Emails were sent on December 3, 2024, with reminders on December 10 and 17. Additionally, the research team phoned brigade adjutants to coordinate further dissemination (WhatsApp messages and in-person work visits). The inclusion period ended December 24.

### Sample Size

We applied the commonly used rule of thumb of at least 10 events per predictor. The primary independent variable, occupational task comprised 13 categories, each treated as a separate predictor in the sample size calculation. In addition, 2 confounders (age and gender) were included. Assuming a weekly health problem prevalence of 40% and a dropout rate of 10%, the required sample size was estimated at 500 participants. Given a source population of approximately 6,500 personnel, this sample size was considered both feasible and sufficient to ensure representativeness.

### Study Procedure

During the 12-week follow-up, participants received a weekly survey every Friday at 09:00 am via a Perfyso pop-up notification. If 2 consecutive questionnaires were missed, an email reminder was sent. The questionnaire was identical each week, except at baseline, week 6, and week 12, when additional items on workability, autonomy, and work support were included (File S1). To facilitate involvement, participants received summaries of their logged data at weeks 3, 6, 9, and 12. Participants were informed that data would not be shared with their employer; however, in case of serious issues requiring immediate medical attention, researchers could notify the staff doctor.

### Survey

#### Demographics

Participants registered their gender, age, and brigade of employment in the baseline questionnaire.

#### Health problems

This study used a modified Dutch-translated version of the OSTRC-H2 to monitor health problems.^8^^,^^10^ The OSTRC-H2 is validated for capturing health problems in sports and has been used previously in Dutch^14^; Bjørneboe et al.^11^ also validated it for military settings. The OSTRC-H2 includes 4 core items assessing a health problem and its impact on performance in the past 7 days, each with 4 response options scored 0, 8, 17, or 25 points. Responses were summed into a severity score (0 = no health problem; 100 = inability to work). Health problems were classified as substantial if item 2 or 3 scored ≥17. Follow-up questions specified whether the problem was a musculoskeletal injury, mental health issue, or illness. Primary outcomes were all health problems (severity index >0) and substantial health problems (item 2 or 3 ≥ 17). Secondary outcomes were all and substantial musculoskeletal injuries, mental health problems, and illnesses (File S1).

#### Workload

The survey contained a single item for physical workload and another for mental workload. Both items were rated on a Borg CR10 scale, ranging from 0 to 10, with 1 representing the lowest workload and 10 representing the highest workload.^15^ However, a similar item has not yet been validated for measuring mental workload.

#### Work ability

The work ability score rates a person’s ability to perform their work in relation to the best period in their life on a scale of 0 (completely unable to work) to 10 “work ability at its lifetime best.”^16^ We reported the work ability score as the mean and standard deviation. Participants registered their work ability at baseline, 6 weeks, and 12 weeks.

#### Occupational tasks

Participants indicated which occupational task they spent most of their time on during the past week from a comprehensive list of all RNLM LTC tasks. Each task had distinct occupational demands, and personnel spent most of their week on one task. Additionally, if participants reported a health problem, they were asked to indicate the occupational task they thought was related to that health problem (File S1).

### Analysis

#### Descriptive statistics

All data processing and statistical analyses were performed using R in RStudio (version 2022.7.0.548).^17^ We reported participants’ ages, gender, and employee type at baseline in means and standard deviations or counts and percentages. For each task, we summarized the number of times it was reported, the number of new health problems, the number of new substantial health problems, and the mean physical and mental workload, and Work Ability Score.

#### Inferential statistics

The second aim of this study was to investigate the relationship between the occupational tasks and the association with new health problems. The relationship between the categorical determinant, namely occupational task, and the dichotomous outcome variable, health problem, was estimated using mixed model analysis with a random intercept for participant ID using the *lme4* package in R.^18^ Considering the ability of mixed model analysis to handle missing values, we took no further steps to account for missingness.^19^ We first estimated a model with only the central determinant (task) and health outcome variable and then a model in which we controlled for age and gender. We reported the results of the uncorrected and corrected models in odds ratios and with 95% confidence intervals.

## RESULTS

### Participants and Response

In total, 346 participants registered for participation in this study through the Perfyso application, of whom 293 completed the first weekly questionnaire including demographic questions. This achieved sample represents 59% of the intended target of 500 participants. Relative to the entire source population of 6,500 personnel, the participation rate was 4.5%. The characteristics of the 293 participants are presented in Table 1. Over the 12-week study period, the mean weekly response rate, based on the 293 participants who started, was 51% (95% CI: 40%-61%). The response rate started at 100% in week 1, dropped to 58% in week 2 and was 41% in week 12 (File S2). The task “Security and Surveillance—transport” was not reported during the study and we combined the 2 Border Policing—Mobile Security Surveillance tasks (car and motorbike) for all further analyses because of limited data and the similarity between these activities (Table 2; File S1). Hence, the final number of analyzed tasks was 12.

**Table 1.** Baseline demographics of the responders in week 1

		Total	Military	Civilian	Reservist
Gender	Female (*n* (%))	66 (23%)	44 (17%)	19 (70%)	3 (60%)
	Male (*n* (%))	220 (75%)	211 (83%)	7 (26%)	2 (40%)
	Prefer not to say (*n* (%))	1 (<1%)	0	1 (4%)	0
	Missing (*n* (%))	6 (2%)			
Age (mean (SD))		39.8 (11.0)	39.9 (10.9)	38.8 (11.9)	42.8 (13.7)

Abbreviations: n, number; SD, standard deviation.

**Table 2.** The number of new health problems per occupational task over a 12-week period

Task	Number of times reported	Number of new health problems (*n* (%))				Number of new substantial health problems (*n* (%))			
		Total	Musculoskeletal	Mental	Illness	Total	Musculoskeletal	Mental	Illness
Deskwork	776	90 (11.6%)	26 (3.4%)	28 (3.6%)	36 (4.6%)	21 (2.7%)	1 (0.1%)	6 (0.8%)	14 (1.8%)
Managing	348	39 (11.2%)	15 (4.3%)	8 (2.3%)	16 (4.6%)	10 (2.9%)	3 (0.9%)	1 (0.3%)	6 (1.7%)
Border policing—airport	172	17 (9.9%)	8 (4.7%)	1 (0.6%)	8 (4.7%)	2 (1.2%)	0 (0.0%)	0 (0.0%)	2 (1.2%)
Border policing—mobile security surveillance	62	12 (19.4%)	5 (8.1%)	2 (3.2%)	5 (8.1%)	2 (3.2%)	0 (0.0%)	1 (1.6%)	1 (1.6%)
Border policing—marine port	55	8 (14.5%)	4 (7.3%)	1 (1.8%)	3 (5.5%)	4 (7.3%)	2 (3.6%)	0 (0.0%)	2 (3.6%)
Security and surveillance—objects	56	7 (12.5%)	1 (1.8%)	2 (3.6%)	4 (7.1%)	6 (10.7%)	0 (0.0%)	2 (3.6%)	4 (7.1%)
Security and surveillance—airport	53	6 (11.3%)	2 (3.8%)	0 (0.0%)	4 (7.5%)	1 (1.9%)	1 (1.9%)	0 (0.0%)	0 (0.0%)
Security and surveillance—subjects	34	7 (20.6%)	5 (14.7%)	0 (0.0%)	2 (5.9%)	3 (8.8%)	2 (5.9%)	0 (0.0%)	1 (2.9%)
Education and instruction	37	6 (16.2%)	2 (5.4%)	2 (5.4%)	2 (5.4%)	1 (2.7%)	0 (0.0%)	1 (2.7%)	0 (0.0%)
International and military police tasks	36	5 (13.9%)	1 (2.8%)	0 (0.0%)	4 (11.1%)	2 (5.6%)	0 (0.0%)	0 (0.0%)	2 (5.6%)
Emergency response	65	8 (12.3%)	3 (4.6%)	2 (3.1%)	3 (4.6%)	1 (1.5%)	0 (0.0%)	0 (0.0%)	1 (1.5%)
Other	165	19 (11.5%)	6 (3.6%)	4 (2.4%)	9 (5.5%)	4 (2.4%)	1 (0.6%)	0 (0.0%)	3 (1.8%)
Overall	1859	224 (12.0%)	78 (4.2%)	50 (2.7%)	96 (5.2%)	57 (3.1%)	10 (0.5%)	11 (0.6%)	36 (1.9%)

All percentages were calculated with the total number of times a task was reported as denominator. Participants reported one task per week.

### Health Problems

In total over the 12-week period 175 (60%) participants reported a total of 224 health problems, of which 96 (43%) were illnesses, 78 (35%) were musculoskeletal injuries, and 50 (22%) were mental health problems. Of the illnesses, 36 (38%) were substantial, of the musculoskeletal injuries, 10 (13%) were substantial, and of the mental health problems, 11 (22%) were substantial (Table 2). The mean weekly prevalence was 23% for all health problems and 5% for substantial health problems.

### Workload and Work Ability

The overall average physical workload was 4.5 out of 10 (SD 2.7). Physical workload was highest for Security and Surveillance—Airport (mean 6.8, SD 3.2) and Border Policing—Marine Port (mean 6.1, SD 2.6), and lowest for Desk Work (mean 3.7, SD 2.3). The overall average mental workload was similar (mean 4.5, SD 2.7), with Managing showing the highest mean mental workload (mean 5.0, SD 2.7) and Border Policing—Mobile Security and Surveillance the lowest (mean 3.6, SD 2.9). The average work ability score for the entire sample was 7.7 out of 10 (SD 1.7), with participants performing Border Policing—Marine Port showing the lowest score (mean 5.9, SD 2.3) (Table 3).

**Table 3.** The number of new health problems, physical and mental workload, and work ability per reported occupational task

Task	Physical workload (mean (SD))	Mental workload (mean (SD))	Work ability (mean (SD))
Deskwork	3.7 (2.3)	4.3 (2.6)	7.6 (1.6)
Managing	5.0 (2.7)	5.0 (2.7)	7.9 (1.5)
Border policing—airport	5.3 (2.6)	4.8 (2.6)	7.7 (1.5)
Border policing—mobile security surveillance	4.6 (2.7)	3.6 (2.9)	7.1 (2.2)
Border policing—marine port	6.1 (2.6)	4.9 (1.8)	5.9 (2.3)
Security and surveillance—objects	4.4 (2.9)	4.6 (2.7)	6.7 (2.1)
Security and surveillance—airport	6.8 (3.2)	4.3 (3.2)	8.1 (1.3)
Security and surveillance—subjects	5.1 (3.1)	4.5 (2.6)	7.2 (3.0)
Education and instruction	4.8 (3.2)	4.7 (2.7)	7.9 (1.2)
International and military police tasks	4.9 (1.7)	4.2 (2.0)	8.1 (1.4)
Emergency response	5.3 (2.5)	4.0 (2.5)	8.1 (0.9)
Other	4.1 (3.1)	4.4 (3.1)	8.0 (1.5)
Overall	4.5 (2.7)	4.5 (2.7)	7.7 (1.7)

### Tasks

The most frequently reported tasks were Deskwork (43%), followed by Managing (19%), and Border Security Task—Airport (9%). We reported the mean physical load, the mean mental load, and the mean work ability score for each occupational task (Table 3).

### Association Between Tasks and Health Problems

The results from the final model, adjusted for age and gender, are presented in Figure 1. During the twelve week study period, there were no statistically significant associations between occupational tasks and new health problems. In general, the more operationally demanding tasks appeared to report more health problems. Among the various tasks, Border Policing—Mobile Security Surveillance, education and instruction, and Security and Surveillance*—*Subjects reported a higher number of health problems compared with other tasks. Specifically, personnel performing Security and Surveillance—Subjects had higher odds of reporting health problems compared to those in Border Policing—Airport (OR: 2.59, 95% CI: 0.98-6.88). Similarly, the task Border Policing—Mobile Security Surveillance had higher odds for health problems compared to both Desk Work (OR: 1.64, 95% CI: 0.83-3.23; Figure 1) and Border Policing—Airport (OR: 2.19, 95% CI: 0.97-4.92; Figure 1). We reported the unadjusted and adjusted models in File S3.

**Figure 1.**
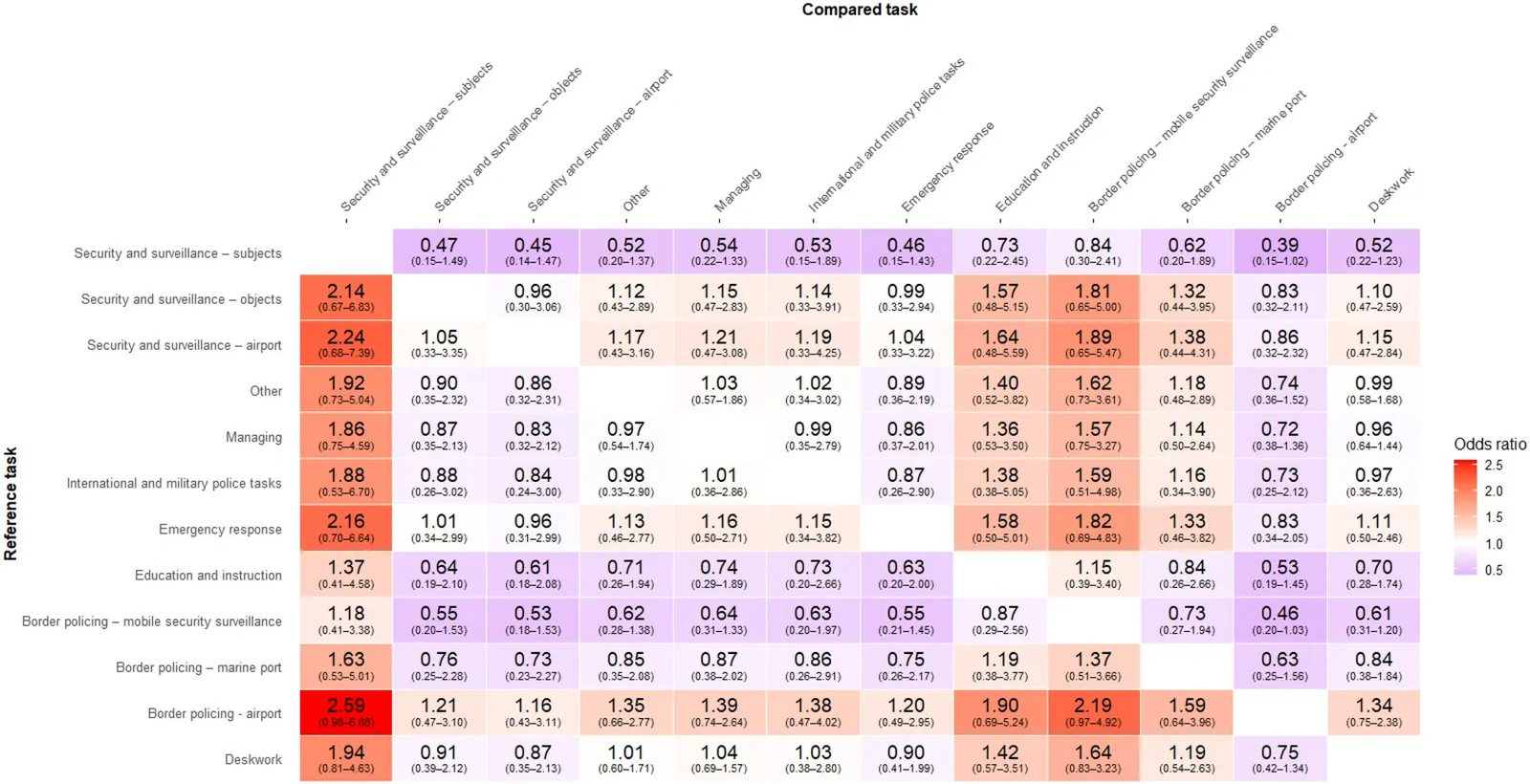
Pairwise odds ratios (or, 95% CI) for reporting any health problem across task categories, estimated with logistic mixed model analysis and adjusted for age and gender. In each tile, the row task is the reference and the column task is the comparator. Warm colors indicate or >1 (higher odds vs. the row task), cool colors or <1. Estimates use weekly primary-task reports (max one task per participant per week). Abbreviations: CI, confidence interval; or, odds ratio.

## DISCUSSION

This study reported the prevalence of health problems and investigated the relationship between occupational tasks and health problems as measured via a weekly surveillance using the OSTRC-H2 questionnaire at the RNLM. We found an average weekly prevalence of health problems of 23% and 5% for substantial health problems. There were no significant associations between occupational tasks and health problems. In general, participants who performed more operationally demanding tasks such as Border Policing—Mobile Security Surveillance and Security and Surveillance—Subjects reported more health problems compared to other occupational tasks. Despite substantial dropout after week 1, response rate remained relatively constant for the remaining weeks. In the final week, 120 participants (41%) were still responding to the weekly surveys, indicating satisfactory adherence among RNLM personnel.

### Compared to Previous Research

To our knowledge, one of the only other studies that used the OSTRC-H2 in a military setting was Bjørneboe et al.,^11^ who found slightly higher prevalences than in our study: 28% vs. 23% for all injuries and 10% vs. 5% for substantial injuries, despite only reporting physical injuries. Additionally, they reported a response rate of 74% which is higher than the response rate in the current study (51%). The main reason for these discrepancies may lie in the type of military personnel that were included in our respective studies. Bjørneboe et al.^11^ included 2 specific companies of a battalion of the Norwegian army, whereas we included soldiers and civilians across the organization. Limiting recruitment to a single, relatively isolated battalion may have improved the response rate. At the same time, the diversity in jobs and associated tasks, including civilians and desk workers, in the current study may have resulted in lower overall prevalences of health problems.

Many studies have investigated risk factors for health problems in a military context,^20^ and several other studies have highlighted the wide range of military occupational tasks and their associated risks.^21^^,^^22^ Nevertheless, this was the first study to investigate the relationship between tasks and health problems in the military. The trend for elevated risk associated with operationally demanding tasks, may be attributed to a combination of factors inherent to these tasks, including prolonged static loading, whole-body vibration while operating vehicles, shift work, and heightened cognitive demands associated with surveillance and human interactions. Previous research has linked such factors to increased rates of musculoskeletal complaints and mental fatigue in military and law enforcement populations.^23-25^

Although several other studies have investigated mental health in military personnel, these studies only included problems that were reported to a medical professional.^26^ Sharp et al.^27^ reported that an estimated 60% of military personnel with mental health problems did not seek help. Social barriers such as not wanting to be perceived as weak, concerns about command’s access to mental health records, and fitness for duty are key deterrents when it comes to reporting adverse mental health in military populations.^28^ A prospective and anonymous monitoring tool, such as the one used in this study, may help overcome some of the existing barriers to mental health reporting. Future research should explore how prospective monitoring compares to conventional health registration in identifying and understanding mental health problems. By doing so, the OSTRC-H2 can also be evaluated as a potential instrument for Worker Health Surveillance, supporting prompt interventions to secure health and fitness of RNLM personnel.

### Strengths and Limitations

This study has several main strengths. To our knowledge, this was one of the first studies that utilized the OSTRC-H2 weekly to capture health problems in an operational military setting. By using the OSTRC-H2 questionnaire, we demonstrated a method for capturing emerging health problems before they result in dropout or limited duty status, thereby providing a meaningful tool for practitioners to use as Worker Health Surveillance Second, because of the prospective nature of this study, we were able to study the temporal association between the occupational tasks and the development of health problems. Finally, this study was conducted in a real-world setting, which enhances the ecological validity and, consequently, the practical applicability of the findings.

Despite these strengths, this study is not without its limitations. The first major limitation of this study is the low recruitment rate in general, and more specifically among operational personnel. This may have resulted healthy-worker bias, as well as selection bias and therefore limit the generalizability of our results. Secondly, while the OSTRC-H2 is a valid and reliable measure for detecting health problems, its self-report nature may have led to reporting bias. In this study, we aimed to counter this source of bias by assuring participants that individual data would in no way be shared with commanders. In this study, we were only able to control for age and gender. There are possibly other confounders in the relation between task and health problems, for example prior health problems, that were not measured in this study. Hence, irrespective of the corrected models, confounding may have influenced our results. Finally, considering the unique occupational demands of RNLM personnel, caution should be taken when generalizing these findings to a broader population.

### Practical Implications

In the military context, occupational demands are often unchangeable. This, of course, is one of the reasons for the strict minimal physical standards characteristic of many military organizations globally. When the nature of the task cannot be adapted, the focus in relation to military readiness shifts toward maintaining and enhancing individual capacity to meet these demands. In this regard, prospective health monitoring or worker health surveillance is crucial for sustaining military readiness.^29^ This study showed that the OSTRC-H2 questionnaire enables the capturing of health problems before they progress to clinical injury or limited duty status.^8^ This early identification offers a window of opportunity for timely intervention by the RNLM and can thereby contribute to reduced absenteeism and overall improved operational readiness. Future research should investigate how the RNLM can support timely interventions based on the results of this monitoring tool.

To achieve the maximum benefit from prospective health monitoring, it must be practically applicable in a military setting. Although this study did not explicitly incorporate implementation outcomes, it revealed important challenges regarding the engagement of personnel performing operational tasks, such as security and surveillance, and border policing. The participation rates among these groups were lower than those of office workers, likely because of limited access to digital systems and operational time constraints. Future research should therefore focus on the adoption of health monitoring among operational personnel. This could include developing alternative delivery methods, such as wearables, adjusting the frequency of questionnaires, and exploring ways to integrate health monitoring into existing workflows.

## CONCLUSION

This study shows that the mean weekly prevalence was 23% for all health problems and 5% for substantial health problems. Response rates remained relatively constant after week 2 thus this monitoring tool may be an effective way to capture health problems before they result in dropout. To improve early detection and support targeted interventions, future research should focus on implementing these monitoring tools in operational settings and integrating its result with current healthcare pathways.

## Supplementary Material

usag174_Supplementary_Data

## Data Availability

Data will be shared upon reasonable request.

## INSTITUTIONAL CLEARANCE

Institutional clearance does not apply.
