# Supplementary material for: Monitoring Health Problems in Military Personnel Using the Oslo Sports Trauma Research Center Questionnaire on Health Problems
Source: Mil Med. 2026 Apr 30;191(11-12):e2552–9. doi: 10.1093/milmed/usag174 (PMC13730449; doi:10.1093/milmed/usag174)
Supplement: Supplementary file 1 [file usag174_supplementary_data.zip › Supplementary file 1.docx]

**Supplementary file 1: weekly survey including items only for baseline, six, and twelve weeks**

|  | Definition | Categories |  |
| --- | --- | --- | --- |
| Question ID |  |  |  |
| *Baseline* | | | |
| I | Participant Identification Number |  |  |
| II | Number of follow-up |  |  |
| A | At which brigade are you employed? | 0 | Brigade Drenthe-IJsselstreek, locatie Darp |
|  |  | 1 | Brigade Oostgrens-Noord |
|  |  | 2 | Brigade Oostgrens-Midden |
|  |  | 3 | Brigade Veluwe |
|  |  | 4 | Brigade Drenthe-IJsselstreek, locatie Groningen |
|  |  | 5 | Brigade Waddengebied |
|  |  | 6 | Brigade Barbant Noord/Limburg Noord |
|  |  | 7 | Brigade Limburg Zuid |
|  |  | 8 | Brigade Brabant Zuid |
|  |  | 9 | Brigade Scheldestromen |
|  |  | 10 | Brigade Grensbewaking |
|  |  | 11 | Brigade Noord-Holland |
|  |  | 12 | Brigade operationele Service & Support |
|  |  | 13 | Brigade Politie & Beveiliging |
|  |  | 14 | Brigade Toezich Beveiliging Burgerluchtvaart |
|  |  | 15 | Brigade Vreemdelingenzaken |
|  |  | 16 | Brigade Recherche |
|  |  | 17 | Brigade Speciale Beveiligingsopdrachten |
|  |  | 18 | Brigade Utrecht |
|  |  | 19 | Brigade Objectbeveiliging |
|  |  | 20 | Brigade Hoog Risico Beveiliging |
|  |  | 21 | Brigade Zuid-Holland |
|  |  | 22 | Brigade Caribisch gebied |
| B | What is your gender? | 0 | Vrouw |
|  |  | 1 | Man |
|  |  | 2 | Non-binary |
|  |  | 3 | Prefer not to say |
| C | What is your age? (years) |  |  |
| *Work ability* | | | |
| D | How would you score your work ability at this time? | 0 | Not able to work |
|  |  | 1 |  |
|  |  | 2 |  |
|  |  | 3 |  |
|  |  | 4 |  |
|  |  | 5 |  |
|  |  | 6 |  |
|  |  | 7 |  |
|  |  | 8 |  |
|  |  | 9 |  |
|  |  | 10 | Work ability at lifetime best |
| *Workload* | | | |
| E | Is your work physically demanding? | 0 | Not physically demanding |
|  |  | 1 |  |
|  |  | 2 |  |
|  |  | 3 |  |
|  |  | 4 |  |
|  |  | 5 |  |
|  |  | 6 |  |
|  |  | 7 |  |
|  |  | 8 |  |
|  |  | 9 |  |
|  |  | 10 | Extremely physically demanding |
| F | Is your work mentally demanding? | 0 | Not physically demanding |
|  |  | 1 |  |
|  |  | 2 |  |
|  |  | 3 |  |
|  |  | 4 |  |
|  |  | 5 |  |
|  |  | 6 |  |
|  |  | 7 |  |
|  |  | 8 |  |
|  |  | 9 |  |
|  |  | 10 | Extremely physically demanding |
| *OSTRC-H2* | | | |
| G | Have you had any difficulties participating work due to injury, illness or other health problems during the past 7 days? | 0 | Full participation without health problems |
|  |  | 1 | Full participation, but with a health problem |
|  |  | 2 | Reduced participation due to a health problem |
|  |  | 3 | Could not participate due to a health problem |
| H | To what extent have you modified your work due to injury, illness or other health problems during the past 7 days? | 0 | No modification |
|  |  | 1 | To a minor extent |
|  |  | 2 | To a moderate extent |
|  |  | 3 | To a major extent |
| I | To what extent has injury, illness or other health problems affected your work during the past 7 days? | 0 | No effect |
|  |  | 1 | To a minor extent |
|  |  | 2 | To a moderate extent |
|  |  | 3 | To a major extent |
| J | To what extent have you experienced symptoms/health complaints during the past 7 days? | 0 | No symptoms/health complaints |
|  |  | 1 | To a mild extent |
|  |  | 2 | To a moderate extent |
|  |  | 3 | To a severe extent |
| K | Which type of health complaint did you experience during the previous week? | 0 | Physical complaint |
|  |  | 1 | Mental complaint |
|  |  | 2 | Illness |
| L | Is this the first time reporting this health problem | 0 | Yes |
|  |  | 1 | No, I reported the same problem last week |
|  |  | 2 | No, I reported the same problem earlier, this was longer than one week ago |
| M | Did you report this health problem to your employer? | 0 | No |
|  |  | 1 | Yes |
|  |  | 2 | Yes and I am receiving medical care for this complaint |
| N | What body region did the physical complaint affect? | 0 | Head/face |
|  |  | 1 | Neck |
|  |  | 2 | Shoulder |
|  |  | 3 | Upper arm |
|  |  | 4 | Elbow |
|  |  | 5 | Forearm |
|  |  | 6 | Wrist/hand |
|  |  | 7 | Finger |
|  |  | 8 | Chest |
|  |  | 9 | Belly |
|  |  | 10 | Upper back |
|  |  | 11 | Lower back |
|  |  | 12 | Pevlis |
|  |  | 13 | Hip/groin |
|  |  | 14 | Thigh |
|  |  | 15 | Knee |
|  |  | 16 | Lower leg |
|  |  | 17 | Ankle |
|  |  | 18 | Foot/toe |
| O | Was this an acute or overuse physical health problem? | 0 | Acute |
|  |  | 1 | Overuse |
| P | Can you explain what happened in your own words? |  |  |
| Q | How would you classify the mental health problem? | 0 | Trouble sleeping |
|  |  | 1 | Stress |
|  |  | 2 | Soberness |
|  |  | 3 | Difficulty concentrating |
|  |  | 4 | Irritable |
|  |  | 5 | Anxious |
|  |  | 6 | Fatigued |
|  |  | 7 | Emotionally unstable |
|  |  | 8 | Reliving traumatic events |
|  |  | 9 | Confused |
|  |  | 10 | Lonely |
|  |  | 11 | Listless |
|  |  | 12 | Worrying |
|  |  | 13 | Reduced self esteem |
|  |  | 14 | Physical complaints without clear physical cause |
| R | Where do you think the mental health problem originated? | 0 | Work life |
|  |  | 1 | Private life |
|  |  | 2 | Both |
| S | Can you explain what happened in your own words? |  |  |
| *Task* | | | |
| T | Which activities did your work mainly consist of during the previous week? (Multiple answers possible) | 0 | Border policing – mobile security surveillance (car) |
|  |  | 1 | Border policing – mobile security surveillance (motorbike) |
|  |  | 2 | Border policing - airport |
|  |  | 3 | Border policing – marine port |
|  |  | 4 | Security and surveillance - objects |
|  |  | 5 | Security and surveillance - subjects |
|  |  | 6 | Security and surveillance - airport |
|  |  | 7 | Security and surveillance - transport |
|  |  | 8 | International and military police tasks |
|  |  | 9 | Deskwork |
|  |  | 10 | Managing |
|  |  | 11 | Education and instruction |
|  |  | 12 | Emergence response |
|  |  | 13 | Other |
| U | From which activities do you think your health problem originated this week? (Multiple answers possible) | 0 | Border policing – mobile security surveillance (car) |
|  |  | 1 | Border policing – mobile security surveillance (motorbike) |
|  |  | 2 | Border policing - airport |
|  |  | 3 | Border policing – marine port |
|  |  | 4 | Security and surveillance - objects |
|  |  | 5 | Security and surveillance - subjects |
|  |  | 6 | Security and surveillance - airport |
|  |  | 7 | Security and surveillance - transport |
|  |  | 8 | International and military police tasks |
|  |  | 9 | Deskwork |
|  |  | 10 | Managing |
|  |  | 11 | Education and instruction |
|  |  | 12 | Emergence response |
|  |  | 13 | Other |
