# Supplementary material for: Monitoring Health Problems in Military Personnel Using the Oslo Sports Trauma Research Center Questionnaire on Health Problems
Source: Mil Med. 2026 Apr 30;191(11-12):e2552–9. doi: 10.1093/milmed/usag174 (PMC13730449; doi:10.1093/milmed/usag174)
Supplement: Supplementary file 1 [file usag174_supplementary_data.zip › Supplementary file 3.docx]

**Supplementary file 2: uncorrected and corrected results from the mixed model analysis for the association between tasks and health problems**

| Uncorrected model | | | | Corrected model | | |
| --- | --- | --- | --- | --- | --- | --- |
|  | Odds ratio (95% CI) | Standard error | p-value | Odds ratio (95% CI) | Standard error | p-value |
| Deskwork (ref) |  |  |  |  |  |  |
| Border policing - airport | 0.84 (0.48–1.45) | 0.28 | 0.53 | 0.75 (0.42–1.33) | 0.29 | 0.32 |
| Border policing - marine port | 1.30 (0.56–2.99) | 0.43 | 0.54 | 1.19 (0.51–2.76) | 0.43 | 0.68 |
| Border policing - mobile security and surveillance | 1.83 (1.26–2.66) | 0.19 | 0.00 | 1.64 (1.08–2.48) | 0.21 | 0.02 |
| Education and instruction | 1.48 (0.63–3.46) | 0.43 | 0.37 | 1.42 (0.61–3.28) | 0.43 | 0.42 |
| Emergency response | 1.07 (0.48–2.38) | 0.41 | 0.87 | 0.90 (0.40–1.99) | 0.41 | 0.79 |
| International and military policing | 1.23 (0.54–2.81) | 0.42 | 0.62 | 1.03 (0.42–2.51) | 0.46 | 0.95 |
| Managing | 0.96 (0.64–1.45) | 0.21 | 0.85 | 1.04 (0.69–1.58) | 0.21 | 0.84 |
| Other | 0.99 (0.60–1.64) | 0.26 | 0.97 | 1.01 (0.61–1.68) | 0.26 | 0.96 |
| Security and surveillance - airport | 0.97 (0.44–2.13) | 0.40 | 0.95 | 0.87 (0.41–1.82) | 0.38 | 0.71 |
| Security and surveillance - objects | 1.09 (0.42–2.85) | 0.49 | 0.86 | 0.91 (0.35–2.35) | 0.49 | 0.84 |
| Security and surveillance - subjects | 1.97 (0.92–4.22) | 0.39 | 0.08 | 1.94 (0.94–4.03) | 0.37 | 0.07 |
| The uncorrected model shows the effect of the occupational tasks on new health problems. The corrected model shows the results adjusted for age and gender. Deskwork was set as the reference category for both models. | | | | | | |
